# Nonvesicular cholesterol transport in physiology

**DOI:** 10.1172/JCI188127

**Published:** 2025-03-17

**Authors:** Alessandra Ferrari, Peter Tontonoz

**Affiliations:** Department of Pathology and Laboratory Medicine, Department of Biological Chemistry, UCLA, Los Angeles, California, USA.

## Abstract

In mammalian cells cholesterol can be synthesized endogenously or obtained exogenously through lipoprotein uptake. Plasma membrane (PM) is the primary intracellular destination for both sources of cholesterol, and maintaining appropriate membrane cholesterol levels is critical for cellular viability. The endoplasmic reticulum (ER) acts as a cellular cholesterol sensor, regulating synthesis in response to cellular needs and determining the metabolic fates of cholesterol. Upon reaching the ER, cholesterol can be esterified to facilitate its incorporation into lipoproteins and lipid droplets or converted into other molecules such as bile acids and oxysterols. In recent years, it has become clear that the intracellular redistribution of lipids, including cholesterol, is critical for the regulation of various biological processes. This Review highlights physiology and mechanisms of nonvesicular (protein-mediated) intracellular cholesterol trafficking, with a focus on the role of Aster proteins in PM to ER cholesterol transport.

## Introduction

**Figure 1:**
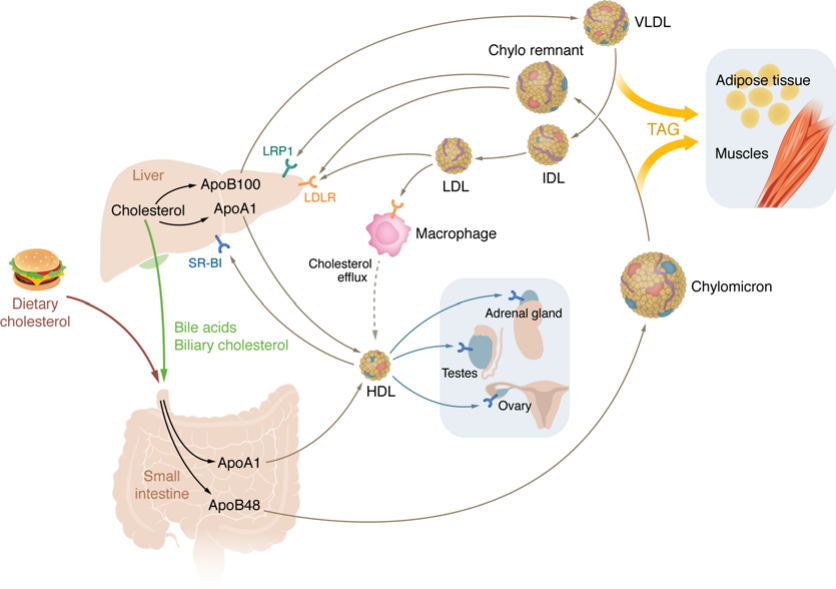
Overview of whole-body cholesterol transport in mice. Diet-derived cholesterol is absorbed by enterocytes in small intestine and incorporated into chylomicrons and HDL. Liver and intestine synthesize cholesterol de novo. Hepatic cholesterol is packaged into VLDL and HDL. Chylomicrons and VLDL are enriched in triglycerides (TAG), which are delivered to periphery tissues, including adipose and muscle. TAG-depleted chylomicrons, called chylomicron remnants, are delivered back to liver, where they bind LRP1 and LDLR. TAG-depleted VLDLs are called intermediate-density lipoproteins (IDLs). When IDLs are further depleted of TAG, they become LDL. LDL is taken up by LDLR in liver and by scavenger receptors in macrophages. Efflux of cholesterol from macrophages to HDL initiates reverse cholesterol transport (RCT) to the liver for excretion. HDL-cholesterol is taken up by hepatic SR-BI in the liver and is converted to bile acids for elimination. In mice HDL is the most abundant lipoprotein and delivers cholesterol to steroidogenic organs. ApoA1, apolipoprotein A-I; ApoB, apolipoprotein B.

Cholesterol is a fundamental component of cellular membranes (1), and is also the precursor of steroid hormones (2), vitamin D (3), oxysterols (4), and bile acids (5). At the whole-body level (Figure 1), cholesterol synthesis contributes to about 70% of daily cholesterol accrual, while 30% of cholesterol derives from diet (6). The liver is the primary site of cholesterol synthesis (6). The liver packages triglycerides and cholesterol esters into very low-density lipoprotein (VLDL), which is then secreted into the circulation (7). VLDL is converted to low-density lipoprotein (LDL) as triglycerides are catabolized by lipoprotein lipase in capillaries of peripheral tissues, including adipose and muscle (8). Liver also produces high-density lipoprotein (HDL) though secretion of apolipoprotein A-I and the transfer of cholesterol by ATP-binding cassette transporter A1 (ABCA1) (9). Most cells in the mammalian body obtain cholesterol primarily from the uptake of HDL or LDL, but can also synthesize cholesterol if necessary from acetyl-CoA (6). Enterocytes in the small intestine are unique in their ability to obtain free cholesterol from the diet and from reabsorption (10). Diet-derived cholesterol in enterocytes is esterified and incorporated together with triglycerides into chylomicrons, which are released into the lymphatics and subsequently reach the circulation. Chylomicrons deliver triglycerides to peripheral tissues, and their remnants are taken up by the liver (Figure 1).

Mammals maintain cholesterol homeostasis through careful coordination of cholesterol synthesis, uptake, conversion, and elimination (11). Two transcription factor families, the liver X receptors (LXRs) and the sterol regulatory element–binding proteins (SREBPs), are particularly important for the coordination of cholesterol metabolism. SREBP2 is the master regulator of all the enzymes needed for cholesterol synthesis and therefore the major determinant of cholesterol production (12). LXRs are critical for the expression of proteins needed for removal of cholesterol from cells, and for reverse transport of cholesterol from the periphery to the liver and then into the gut lumen (13). Cholesterol cannot be catabolized by mammalian cells and must be excreted directly into bile or converted to bile acids to be removed from the body. SREBP2 activity is responsive to endoplasmic reticulum (ER) membrane cholesterol concentrations, while LXRs sense cholesterol through the binding of oxysterol ligands.

## The ER’s central role in controlling cholesterol homeostasis

Most cholesterol in mammalian cells resides in the plasma membrane (PM), where it accounts for about 40 mol% of all lipids (14). In the ER, cholesterol represents less than 5 mol%, or about 1% of total cellular cholesterol (15, 16). Despite being relatively cholesterol-poor, the ER plays a fundamental role by serving as the intracellular cholesterol sensor (17). The ER must closely monitor PM cholesterol levels to ensure cellular cholesterol homeostasis. This regulation involves the controlled transport of a distinct fraction of cholesterol, referred to as “accessible cholesterol” or chemically active cholesterol, from the PM to the ER. In the PM, the majority of cholesterol forms complexes with sphingomyelin and other phospholipids, rendering it “inaccessible” or chemically inactive for transport (18–21).

Phospholipid and sphingolipid species vary in their ability to form complexes with cholesterol (22–24). As a result, the accessibility of cholesterol is influenced, in part, by membrane phospholipid composition. Cholesterol exhibits the strongest affinity for sphingomyelin and other sphingolipids, followed by phospholipids with saturated fatty acyl chains, while it forms weaker complexes with unsaturated phospholipids (25, 26). The outer leaflet of the PM is rich in sphingomyelin and saturated phosphatidylcholine, enabling it to retain cholesterol effectively (27). Approximately 15 mol% of total PM lipids consist of cholesterol bound to sphingomyelin, rendering this cholesterol inaccessible for transport. Cholesterol accessibility in a membrane is minimal when cholesterol levels are low and does not increase significantly until a threshold — determined by the phospholipid content — is reached. Once this threshold is exceeded, cholesterol accessibility rises sharply (28). Under normal physiological conditions, the pool of accessible cholesterol remains a small fraction of the total membrane cholesterol, as it is quickly transported or modified (19).

When PM cholesterol surpasses a certain threshold, the expanded pool of accessible cholesterol is quickly trafficked to other organelles, including the ER. The increase in ER cholesterol levels then inhibits the cleavage of SREBP2, thereby reducing cholesterol biosynthesis and uptake to prevent excess accumulation and ensure proper PM cholesterol levels (19, 29, 30). The movement of cholesterol to the ER is also required for the esterification of cholesterol. ER-resident acyl-CoA:cholesterol acyltransferase (ACAT) enzymes facilitate the production of cholesterol esters that can be stored in intracellular lipid droplets or secreted in lipoproteins (31–33). In hepatocytes, the transfer of cholesterol to the ER also facilitates the conversion of cholesterol into oxysterols or bile acids (34).

## Overview of nonvesicular cholesterol trafficking

Because of its hydrophobic nature, cholesterol rapidly moves between the two leaflets of the phospholipid bilayer (35). Cholesterol is unable to diffuse through the cytosol, however, and therefore cells must engage specific mechanisms of transport to allow its movement between different organelles. Vesicular mechanisms play an important role in lipid movement, and these are reviewed elsewhere (36). Here we will focus on nonvesicular (protein-mediated) pathways.

**Figure 2:**
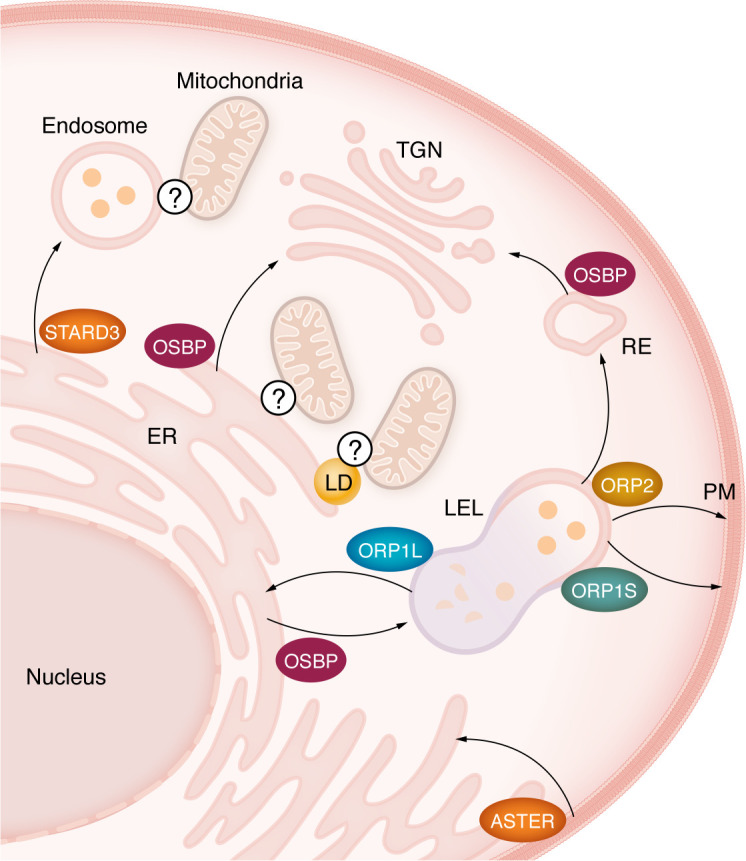
Membrane contact sites are involved in nonvesicular cholesterol trafficking. OSBP facilitates the transport of cholesterol from the ER to the *trans*-Golgi network (TGN) in exchange for phosphatidylinositol 4-phosphate (PI4P). OSBP also mediates cholesterol transport from the ER to the late endosomes/lysosomes (LELs) and from recycling endosomes (REs) to the TGN. ORP1S and ORP2 mediate the transport of cholesterol from LELs to the plasma membrane (PM). ORP1L supports endosomal sorting and forms ER–LEL/multivesicular body tethers, and was proposed to be a mediator of cholesterol trafficking between these organelles. STARD3 transports cholesterol from ER to endosomes. Aster proteins form PM-ER contact sites upon cellular cholesterol loading and move the PM pool of accessible cholesterol to the ER. Mechanisms of nonvesicular cholesterol trafficking to mitochondria through lipid droplet–mitochondria (LD-mitochondria), ER-mitochondria, and endosome-mitochondria contact sites have been postulated. Further studies are needed to elucidate which proteins mediate cholesterol trafficking at these membrane contact sites.

In recent years, several mechanisms of nonvesicular lipid transport have been described (Figure 2). Nonvesicular trafficking between membranes is facilitated by lipid transfer proteins and occurs at membrane contact sites (MCSs), where membranes are closely tethered (37, 38). Cholesterol transport by lipid transfer proteins is often coupled with the transport and metabolism of other lipids, particularly phosphoinositides (39), phosphatidylserine, and sphingolipids (40). Cholesterol trafficking between cellular membranes ensures the maintenance of a physiological/optimal level of cholesterol in different organelles. For example, cholesterol is relatively abundant in the *trans*-Golgi network (TGN) and recycling endosomal compartments, and levels decrease toward the *cis*-Golgi (41).

## Oxysterol-binding proteins

Since the ER is also the site where the endogenous synthesis of cholesterol occurs, efficient mechanisms for cholesterol export must be in place to avoid the toxic accumulation of cholesterol in the ER membrane. Studies in yeast (42) and mammalian cells (43) demonstrated that blocking vesicular transport of newly synthesized cholesterol from ER to PM had minimal effect on the appearance of cholesterol at the PM. This suggested that nonvesicular trafficking must be involved, and led to the characterization of the role of a family of proteins named oxysterol-binding proteins (OSBPs) and OSBP-related proteins (ORPs) (44). OSBP is involved in the trafficking of cholesterol from ER to TGN in exchange for phosphatidylinositol 4-phosphate (PI4P) (45). It contains an N-terminal pleckstrin homology (PH) domain responsible for the binding of PI4P, a central FFAT motif that binds the ER membrane protein VAP, and a C-terminal lipid transport domain (46). OSBP1 is tethered at the TGN, not only by the interaction of the PH domain with PI4P, but also by interaction with the small G protein Arf1-GTP (47). OSBP also mediates cholesterol transport from the ER to the limiting membrane of lysosomes by localizing at ER-lysosome contacts, thus activating mTORC1 (48). OSBP, by interaction with RELCH (a Rab11 effector adaptor protein), was reported to be a mediator of cholesterol trafficking from recycling endosomes (REs) to the TGN (49).

## OSBP-related proteins

OSBP-related protein 1 (ORP1) and ORP2 have also been implicated in nonvesicular trafficking of cholesterol. Two splicing variants of ORP1, ORP1S (short isoform, containing only the C-terminal lipid-binding domain) and ORP1L (long isoform, containing the ankyrin repeats, the PH domain, the FFAT motif, and the lipid-binding domain), have been described (50). Studies in HeLa cells lacking ORP1 (51) and HEK293 cells lacking ORP2 (52) demonstrated that ORP1S and ORP2 are responsible for movement of cholesterol from late endosomes/lysosomes (LELs) to PM. The ORP2-dependent deposition of cholesterol into the PM is coupled with the removal of phosphatidylinositol 4,5-bisphosphate [PI(4,5)P_2_] (52). The longer isoform of ORP1, ORP1L, localizes both to LELs and to the multivesicular endosomes/bodies (MVBs), important for endosomal sorting (53). The FFAT motif of ORP1L mediates ER–LEL/MVB contacts by binding ER-localized VAP (54, 55), although it is still unclear whether ORP1L mediates cholesterol transport at these contact sites or whether it only provides tethers (56). Finally, it has been reported that the cholesterol transport activity of the C-terminal lipid-binding domain of ORP1 is strongly enhanced by PI(4,5)P_2_ and phosphatidylinositol 3,4-bisphosphate [PI(3,4)P_2_], suggesting a possible role for ORP1S and ORP1L in mediating cholesterol trafficking at membranes enriched in these phospholipids, such as PM and LELs.

## START proteins

Another class of sterol transporters that have been extensively studied in mammalian cells is the steroidogenic acute regulatory protein–related (StAR-related) lipid transfer (START) protein family (57). The 15 members of this family are defined by a StAR domain (STARD) consisting of an α-helix/β-grip fold that forms an inner tunnel wide enough to accommodate hydrophobic lipids (58). Five StAR proteins have been reported to bind and transport cholesterol. STARD1 moves cholesterol from the outer mitochondrial membrane to the inner membrane in steroidogenic cells (59). STARD3 and STARD3 N-terminal like (STARD3NL) transport cholesterol at MCSs between late endosomes and the ER. Thanks to the presence of an FFAT motif, these two proteins can interact with the ER-anchored VAP proteins (60). STARD4, STARD5, and STARD6 share approximately 30% amino acid identity with one another, and contain only the START domain (61).

Among the family of START proteins, recent findings suggest a role of STARD5 in intracellular cholesterol movement. In murine-derived cells, STARD5 expression is induced by ER stress (62), and was proven to be able to transfer cholesterol between membranes (63). STARD5 is expressed in macrophages and in the liver, and it has been shown that STARD5 deletion in macrophages reduced PM cholesterol content, with significant reduction in the pool of accessible cholesterol, and this correlates with increased PM fluidity. STARD5 overexpression in CHO cells was instead reported to expand the accessible cholesterol pool, suggesting a role in the flux of cholesterol to the PM. STARD5 deletion in macrophages also caused an increase in ACAT activity and cholesterol esterification, and a higher efflux of cholesterol. In hepatocytes, ER stress expanded the pool of membrane-accessible cholesterol in a STARD5-dependent manner. Liver of STARD5-deficient mice recapitulated the phenotype of STARD5^–/–^ macrophages with increased cellular cholesterol and ACAT activity. Moreover, STARD5-deficient mice fed a Western diet were reported to have impaired VLDL secretion and hepatic cholesterol accumulation (64), supporting the involvement of STARD5 to deliver cholesterol to the PM for efflux.

Among the members of the START family, STARD4 is the only one that has been suggested to transfer cholesterol from the PM to the ER or REs. The role of STARD4 in the movement of cholesterol from the PM to the ER and REs (65, 66) is supported by cell biology studies. The presence of a surface-exposed basic patch in STARD4 favors interaction with membranes enriched with anionic lipids, and allows cholesterol to diffuse into the sterol-binding pocket (67). *Stard4* is transcriptionally regulated by SREBP2 in the liver (68), implying that cholesterol depletion drives the expression of the transcript. SREBP2-mediated regulation requires the processing of SREBP2, and therefore a drop in the levels of cholesterol in the ER. In this light, it is unexpected that lower cholesterol levels in the cell would activate a pathway that would eventually dilute cholesterol in the membrane. STARD4-mediated cholesterol transport could be a cellular response required to prevent an excessive drop in ER cholesterol, rather than a primary route to deliver exogenous cholesterol from PM to ER. In line with this idea, the analysis of murine models has not yet linked deletion of STARD4 to alterations in plasma or tissue cholesterol (69). This suggests that STARD proteins may have redundant functions with each other or with members of other sterol transport families.

## Aster proteins

In recent years an additional family of proteins, named Asters (Greek for “star”), have emerged as key players in nonvesicular cholesterol trafficking in mammalian cells (70). Importantly, three laboratories have now independently confirmed the function of Aster proteins in cholesterol transport from PM to ER (70–72). This family includes three closely related proteins, Aster-A, Aster-B, and Aster-C (encoded by the *Gramd1a–c* genes in mice). Asters possess an N-terminal Rab-like GTPase activators and myotubularins (GRAM) domain, a central START-like (ASTER) domain, and a C-terminal single-pass transmembrane domain that anchors them in the ER.

The GRAM domain is structurally similar to the PH domain, which is often present in lipid transfer proteins and binds anionic lipids, including phosphatidylinositol phosphates (PIPs) (73, 74). When expressed in cultured cells, the soluble Aster GRAM domain localizes to the cytoplasm. However, loading of cells with cholesterol induces robust recruitment of the GRAM domain to the PM (70). On the other hand, a truncated form of Aster-B lacking the GRAM domain is unable to move to the PM in response to cholesterol loading (70). Further studies elucidated that the recruitment of the Aster GRAM domain to PM-ER contact sites requires both a sufficient pool of accessible cholesterol and the presence of anionic phospholipids including phosphatidylserine (70, 71, 75). Phosphatidylserine is enriched into the cytosolic leaflet of the PM, and it has been proposed that it could retain cholesterol to this layer (76). Treatment of cells with sphingomyelinase (SMase), which releases accessible cholesterol, also promotes GRAM domain recruitment to the PM (71).

Ercan and collaborators proposed that a basic patch on the GRAM domain is required for the recognition of the acidic head of anionic lipids in the PM. They identified two residues in the GRAM domain, K161 and R191, whose mutation reduces binding to liposomes (77). Another evolutionarily conserved residue in the GRAM domain, R189, was shown to affect the binding at the membrane. The point mutation R189W has been associated with intellectual disability in humans (78, 79), and was reported to bind less efficiently to liposomes containing cholesterol (77).

The ASTER domain of Aster-A, -B, and -C shows structural similarities to the sterol-binding START domain proteins despite minimal primary sequence conservation (58). This domain binds cholesterol and select oxysterols, thereby mediating their transfer between membranes in vitro (70, 71). The crystal structure of the ASTER domain of Aster-A in complex with 25-hydroxycholesterol shows a highly curved 7-stranded β-sheet that forms a cavity to accommodate the sterol ligand. The cavity is closed by an extended carboxy-terminal helix and two shorter helices after the amino-terminal β-strand, with a small opening between β-strands 3 and 4, which allows sterols to access the pocket (70).

## Transcriptional regulation of Asters

The *Gramd1b* gene encoding Aster-B is a target for regulation of liver X receptors (LXRs), a connection that facilitated its initial identification as a sterol-binding factor. LXRs, whose endogenous ligands are oxysterols and intermediates in cholesterol biosynthesis (reviewed in ref. 13), play key roles in the regulation of lipid metabolism. They control multiple steps in reverse cholesterol transport, including induction of the ABCA1 efflux transporter in peripheral cells. LXRs regulate cholesterol excretion from the liver by transcriptionally activating the gene encoding cytochrome P450 7A1 (*Cyp7a1*), the rate-limiting enzyme of bile acid synthesis, and the ATP-binding cassette (ABC) transporters ABCG5 and ABCG8, which are responsible for the transport of cholesterol into the bile (80). *Gramd1b* is highly induced in macrophages in response to LXR activation by synthetic ligands, while all three Aster transcripts are induced in the small intestine upon oral administration of the LXR agonist GW3965. These findings establish Aster-mediated nonvesicular cholesterol transport as another component of the regulatory network controlled by LXRs.

Recent findings also showed that *Gramd1c* is transcriptionally induced in both murine liver and primary hepatocytes by the nuclear receptor FXR (81). Chromatin immunoprecipitation–sequencing data revealed FXR binding to the promoter region of Aster-C in the liver but not in the ileum (82). These findings indicate that the transcriptional regulation of Asters is orchestrated differently across various tissues and organs, suggesting tissue-specific modulation of nonvesicular cholesterol trafficking. Tissue-specific regulation of Aster proteins by nuclear receptors likely reflects the distinct roles of sterol transport in various physiological processes.

## Asters in steroidogenesis

Cholesterol transported by HDL particles containing apolipoprotein A-I primarily enters cells through scavenger receptor class B type I (SR-BI) (83). Most of the HDL-cholesterol is delivered by reverse cholesterol transport from peripheral tissues to the liver and steroidogenic tissues. In the liver, HDL-derived cholesterol can be secreted into bile or excreted through conversion into bile acids (84), while in the steroidogenic organs it serves as a precursor for the synthesis of steroid hormones (85). SR-BI is abundantly expressed on the PM of hepatocytes and steroidogenic cells. Structural studies suggest that SR-BI possesses a cavity that forms a tunnel through which cholesterol is delivered from the lipoprotein to the outer leaflet of the PM (86). In steroidogenic organs, cholesterol derived from HDL is stored as cholesterol esters in lipid droplets (87). These cholesterol ester stores can be mobilized as needed for the synthesis of steroid hormones through the action of hormone-sensitive lipase (88, 89).

The mechanisms underlying the movement of HDL-cholesterol downstream of SR-BI from the PM to the ER were previously unknown. The identification of the Asters sheds new light on this pathway. Aster-B is highly expressed in cortical cells of the adrenal gland, which are specialized to synthesize corticosteroids (70). In vivo studies showed that genetic deletion of Aster-B dramatically reduced the normally abundant cholesterol ester stores in the adrenal cortex. Cholesterol esterification takes place in the ER and is mediated by acyl-CoA:cholesterol acyltransferase 1 (ACAT1) in steroidogenic tissues. Interestingly, Aster-B deletion recapitulates phenotypes previously described for mice lacking SR-BI (90) or ACAT1 (91). This finding strongly implies that the three proteins are part of one axis that ensures efficient cholesterol ester storage. As an additional consequence of impaired PM-ER cholesterol trafficking in the adrenal gland, SREBP2 target genes were potently induced upon Aster-B deletion. This observation suggests an effort by the cells to increase cholesterol synthesis to compensate for loss of HDL-mediated uptake. Despite this increase, however, mice lacking Aster-B still exhibit impaired glucocorticoid production under the stress of fasting (70).

Another tissue expressing particularly high levels of Aster-B is the ovary, the main site of estrogen production (92). Estrogen deficiency is linked to an increased risk of obesity and metabolic disorders in women (93), and menopausal hormonal decline correlates with the accumulation of visceral fat and increased cardiovascular risk (94). Genetic ablation of Aster-B in female mice led to increased body weight gain and hypercholesterolemia during Western diet feeding — a phenotype comparable to that observed in ovariectomized mice (95). Loss of Aster-B was further shown to impair nonvesicular trafficking of cholesterol in ovaries, leading to hypogonadism and reduced estradiol synthesis. Administration of exogenous estradiol partially attenuated the diet-induced obesity induced by Aster-B deficiency.

## Asters in hepatic metabolism

The liver is a major site for HDL-cholesterol uptake. Hepatic intracellular trafficking of cholesterol from PM to ER is essential for many pathways involved in systemic metabolism.

The hepatocyte ER is not only the site of SREBP2 regulation and cholesterol esterification (31), but it also houses machinery necessary for the production of important products of cholesterol, and its conversion into oxysterols (96) and bile acids (97). Both Aster-A and Aster-C were found to be highly expressed in hepatocytes (81), and localization studies showed that Aster-C moves to PM in response to cholesterol loading of primary hepatocytes. Analysis of the function of Asters in hepatic metabolism required the generation of a mouse with liver deletion of both Aster-A and -C (98). Aster-A/C–deficient hepatocytes showed expansion of the pool of accessible cholesterol at the PM, presumably because of impaired cholesterol transfer to the ER.

**Figure 3:**
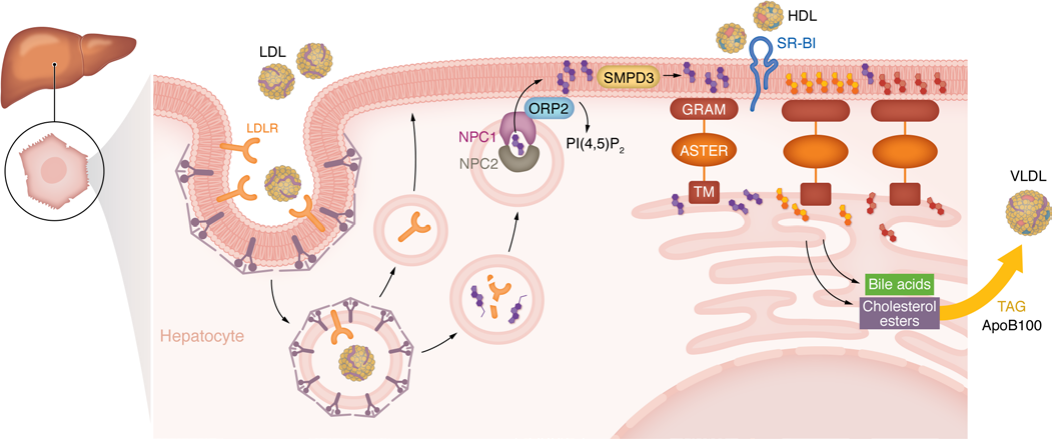
Aster-mediated trafficking of cholesterol in hepatocytes. In hepatocytes, Aster-A and Aster-C move PM cholesterol to the ER. The accessible cholesterol available for intracellular trafficking comes from the release of sphingomyelin-sequestered cholesterol, resulting from SMase (SMPD3) activation during fasting, or from deposition into the PM of lipoprotein-derived cholesterol. Both HDL- and LDL-cholesterol is taken up by hepatocytes. LDL particles, after binding LDLR in coated pits, are internalized in coated vesicles that rapidly fuse with lysosomes. LDLR separates from LDL and is recycled in recycling endosomes (REs) for transport to the PM. Receptors that are not separated from LDL are degraded with the lipoprotein in the lysosome, where cholesterol esters are hydrolyzed to free cholesterol. Through NPC1/NPC2 and ORP2, lysosomal cholesterol is deposited in the PM. The higher content of accessible cholesterol in the PM engages Aster proteins, thus favoring the trafficking of cholesterol to the ER. In the hepatocyte ER cholesterol is converted into bile acids or esterified before incorporation into VLDL. TM, transmembrane.

Analysis of mice with hepatic Aster-A/C knockout revealed two physiological processes that generate accessible PM cholesterol and engage the Aster pathway in liver in vivo: fasting and reverse cholesterol transport (RCT). Prolonged fasting inhibits lipogenesis (99), but at the same time promotes accumulation of triglycerides (100) and cholesterol esters (81). The increase in cholesterol esters correlates with reduced PM cholesterol, implying that mobilization of accessible PM cholesterol sustains fasting-induced esterification. Xiao et al. showed that fatty acids released from adipose tissue during fasting promote the hydrolysis of sphingomyelin, whose content in the liver PM is also reduced after 16 hours of fasting (81). Sphingomyelin degradation allows the release of sequestered cholesterol, resulting in an expansion of the pool of accessible cholesterol available for transport to ER by Asters (Figure 3). Hepatic Aster deletion reduced cholesterol esters accumulation during fasting and consequently lowered liver VLDL-TG secretion. These findings indicate that nonvesicular cholesterol trafficking from PM to ER sustains hepatic lipid output during fasting (81).

The hepatocyte PM-accessible cholesterol pool also expands during RCT. SR-BI in hepatocytes loads cholesterol from HDL into the PM, increasing the accessible pool (Figure 3). Tracer studies with HDL containing ^14^C-labeled cholesterol showed that Aster deficiency reduced incorporation of ^14^C into cholesterol esters, indicating impaired cholesterol transport from the PM to the ER. Furthermore, mice with reduced Aster PM-ER cholesterol transport exhibited decreased movement of HDL-derived cholesterol into bile and decreased conversion into bile acids. These results show that SR-BI binding to HDL makes PM cholesterol available for nonvesicular transport to the ER and establish a role for the hepatic Aster pathway in the final steps of RCT.

## Intersection of vesicular and nonvesicular LDL-cholesterol movement

Cholesterol in LDL is delivered to cells via LDL receptors on the PM (101). The LDL receptor is internalized by endocytosis in coated vesicles and delivered to lysosomes (102), where cholesteryl esters from LDL are hydrolyzed (103). Multiple routes for post-lysosomal cholesterol movement have been hypothesized (104), including (a) transport from late endosomes to sterol-rich membranes (e.g., Golgi), followed by retrograde transport to the ER (105); (b) direct transfer of cholesterol from late endosomes to the ER, possibly through lipid transfer at MCSs (106); and (c) trafficking of cholesterol from lysosome to the PM prior to delivery to the ER and other compartments (107–109). Recent findings have supported the importance of the third hypothesis and revealed the cooperation of vesicular and nonvesicular mechanisms in the delivery of LDL-derived cholesterol to the ER (Figure 3).

Free cholesterol is exported from the lumen of late endosomes/lysosomes through a series of steps involving Niemann-Pick C2 (NPC2) and Niemann-Pick C1 (NPC1) (110, 111). OSBPL2/ORP2 has been implicated in lysosomal cholesterol movement to the PM (52, 112). While it is possible that a fraction of LDL-derived cholesterol can be directed to the ER from lysosomes, experimental evidence indicates that LDL-cholesterol is preferentially deposited into the PM before being transferred to the ER (113). Recent studies have further shown that the mobilization of LDL-derived cholesterol from PM to ER is mediated by Asters. Genetic deletion of Asters (114) or Aster inhibition by a small molecule (115) causes accumulation of LDL-cholesterol in the PM, depletion of cholesterol at the ER, and activation of the SREBP2 pathway. Tracer studies with LDL containing ^14^C-labeled cholesterol in mice with hepatic deletion of Aster-A and -C showed impaired incorporation of ^14^C in cholesterol esters, confirming the involvement of Asters in physiological trafficking of LDL-cholesterol in the liver (81) (Figure 3).

## Asters in dietary cholesterol uptake

The absorption of dietary lipids takes place mainly in the proximal small intestine. In the intestinal lumen, cholesterol is emulsified by bile salts and incorporated along with fatty acids into micelles (116). After cholesterol enters enterocytes, it moves to the ER, where it can be esterified by ACAT2 (117, 118) and incorporated into chylomicrons for systemic absorption. Niemann-Pick C1–like 1 (NPC1L1) has a critical role in the initial phase of cholesterol uptake into the enterocyte PM (119). However, the fate of cholesterol downstream of its uptake into PM of enterocytes has remained unclear. Recent work has described a role for Aster-B and Aster-C in mediating the nonvesicular trafficking of diet-derived cholesterol from PM to ER in the small intestine.

*Gramd1b* and *Gramd1c* are highly expressed in differentiated enterocytes, while *Gramd1a* is prevalent in other intestinal cell types, including crypt stem cells and T cells (120). Intestinal deletion of Aster-B and -C results in lower uptake of diet-derived cholesterol into enterocytes and into the circulation, and attenuates diet-induced hypercholesterolemia (120). Importantly, the absorption of fatty acids and glucose is preserved in the absence of Asters, indicating a specific role in cholesterol handling. Mice lacking intestinal Asters maintain the capacity to assemble chylomicrons, but their chylomicrons have reduced cholesterol content. Studies in intestinal enteroids showed that Aster ablation impairs the trafficking of cholesterol from the apical PM to the ER. As a result, the pool of accessible cholesterol at the membrane of enterocytes expands, ER cholesterol is depleted, and the SREBP2 pathway for cholesterol synthesis is induced.

Functional NPC1L1 is essential to make dietary cholesterol available for transfer to the ER and for its subsequent incorporation into chylomicrons for absorption. Structural biology studies have helped elucidate the mechanism by which NPC1L1 mediates cholesterol entry and membrane deposition in enterocytes (121–123). Cryogenic electron microscopy experiments showed that the N-terminal domain of NPC1L1 possesses a cavity that can bind cholesterol, and that the binding of cholesterol makes the domain mobile. Rotation of the domain forms a tunnel that allows cholesterol to enter the membrane. Cell biology studies have shown that NPC1L1 is internalized via clathrin-dependent endocytosis when enterocytes are exposed to dietary cholesterol (61). The clathrin adaptor Numb binds the C-terminal domain of NPC1L1 and recruits AP2 and clathrin (124). NPC1L1 colocalizes with Rab11 after oral cholesterol administration (125), indicating that NPC1L1 endocytic vesicles are incorporated into the endocytic recycling compartment (ERC). More recently, LIM domain and actin-binding 1 (LIMA1) has been identified as a regulator of intestinal absorption owing to its ability to facilitate NPC1L1 recycling (126). These findings support the importance of NPC1L1 recycling for the absorption of dietary cholesterol. However, whether cholesterol reaches the ER via NPC1L1-mediated ERC has not been established.

**Figure 4:**
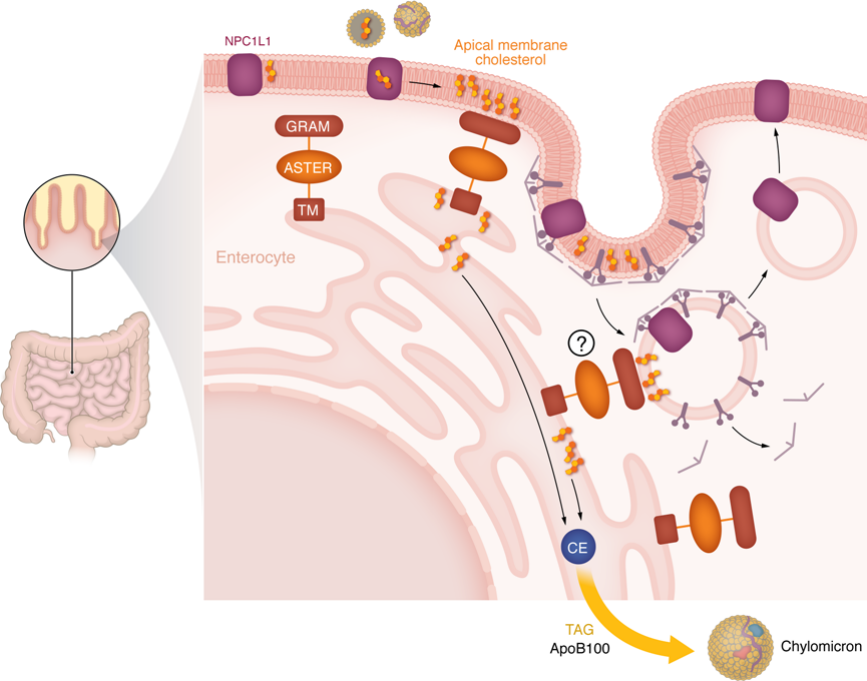
Aster-mediated trafficking of cholesterol in enterocytes. NPC1L1 controls the first step of the process of dietary cholesterol absorption, by gating diet-derived cholesterol uptake by enterocytes. NPC1L1 is present at the apical membrane of the enterocytes when the concentration of cholesterol is low, and it allows the entry of cholesterol and deposition in the lipid bilayer. This expands the pool of accessible cholesterol at the apical membrane and engages Aster-B and Aster-C. NPC1L1 is internalized in clathrin-coated vesicles, recycled in the endocytic recycling compartment (ERC), and redirected to the membrane. The movement of cholesterol from PM to ER allows cholesterol esterification and incorporation onto chylomicrons for systemic absorption. It has been speculated that Asters may also be recruited to cholesterol-enriched ERC, but this requires further study. Aster-mediated trafficking of cholesterol to the ER decreases the level of cholesterol in the PM, thus favoring NPC1L1 recycling.

Our work suggests that Asters work in concert with NPC1L1 (Figure 4) to allow dietary cholesterol to rapidly reach the enterocyte ER. The saturation of the apical PM with cholesterol by NPC1L1 is necessary to recruit Aster-B and Aster-C to the apical PM of enterocytes (120). Genetic deletion of NPC1L1 or inhibition by ezetimibe blocks Aster recruitment to the PM by cholesterol in vivo, positioning NPC1L1 upstream of the Aster pathway. Genetic deletion of Asters impairs cholesterol movement to the ER, even when NPC1L1 is present. Thus, NPC1L1 alone cannot fully explain cholesterol movement to the ER in enterocytes. This model for cooperation between NPC1L1 and Asters does not rule out that some cholesterol may be incorporated into endosomes and ERC along with NPC1L1, and from there undergo vesicular transfer to the ER (Figure 4). Additionally, it seems likely that other proteins participate in nonvesicular or vesicular transport in enterocytes, as cholesterol absorption is not completely abrogated in Aster-knockout models.

Pharmacological inhibition of NPC1L1 by the hypocholesterolemic drug ezetimibe is highly effective at blocking cholesterol absorption. Interestingly, Aster-mediated nonvesicular trafficking in intestine is also pharmacologically targetable. The small-molecule inhibitor AI-3d mimics the effects of Aster genetic deletion on cholesterol movement in enterocytes (120). In murine and human enteroids, treatment with AI-3d expands the pool of accessible cholesterol in the PM. Moreover, oral administration of AI-3d to mice reduces cholesterol trafficking to the ER and cholesterol absorption. The Aster pathway may offer an additional opportunity for pharmacological manipulation of dietary cholesterol uptake.

## Potential involvement of Asters in other lipid transport

Aster proteins have been proposed to facilitate the trafficking of cholesterol (41, 42) and carotenoids (43) at mitochondria-ER contacts based on cell culture studies. However, analysis of mice lacking one or more Aster proteins has not yet revealed phenotypes consistent with these proposed functions. Asters localize specifically to PM in response to cholesterol loading in cell models and in vivo. Furthermore, the phenotypes of Aster-deficient mice described to date can be explained by defects in PM to ER transport. Additional studies of in vivo models will be required to fully define contributions of Aster to cholesterol transport in various physiological contexts.

## Conclusions

Studies aimed at understanding nonvesicular cholesterol transport mechanisms and the biology of Aster proteins have provided new insights into cholesterol homeostasis. Asters play a pivotal role in cholesterol transfer between the PM and ER in many if not all cell types, and are emerging as key regulators in lipid metabolism. Despite this progress, much remains to be explored. The prominent expression of Asters in various tissues, including the heart, skeletal muscle, brain, and immune cells, suggests that additional functions remain to be described. Furthermore, the observation that alternative vesicular or nonvesicular pathways partly compensate for the absence of Aster proteins points to the complexity of cellular cholesterol transport. Further research will be crucial to fully unravel cholesterol trafficking mechanisms and their potential therapeutic implications in diseases linked to perturbations of cholesterol homeostasis.

## Version 1 03/17/2025

Electronic publication

## References

[1] Maxfield FR, Tabas I (2005). Role of cholesterol and lipid organization in disease. Nature.

[2] Miller WL (1988). Molecular biology of steroid hormone synthesis. Endocr Rev.

[3] Prabhu AV (2016). Cholesterol-mediated degradation of 7-dehydrocholesterol reductase switches the balance from cholesterol to vitamin D synthesis. J Biol Chem.

[4] Schroepfer GJ (2000). Oxysterols: modulators of cholesterol metabolism and other processes. Physiol Rev.

[5] Russell DW (2003). The enzymes, regulation, and genetics of bile acid synthesis. Annu Rev Biochem.

[6] Dietschy JM (1984). Regulation of cholesterol metabolism in man and in other species. Klin Wochenschr.

[7] Smith LC (1978). The plasma lipoproteins: structure and metabolism. Annu Rev Biochem.

[8] Goldberg IJ (1996). Lipoprotein lipase and lipolysis: central roles in lipoprotein metabolism and atherogenesis. J Lipid Res.

[9] Sahoo D (2004). ABCA1-dependent lipid efflux to apolipoprotein A-I mediates HDL particle formation and decreases VLDL secretion from murine hepatocytes. J Lipid Res.

[10] Iqbal J, Hussain MM (2009). Intestinal lipid absorption. Am J Physiol Endocrinol Metab.

[11] Brown MS (2018). Retrospective on cholesterol homeostasis: the central role of Scap. Annu Rev Biochem.

[12] Sakai J (1996). Sterol-regulated release of SREBP-2 from cell membranes requires two sequential cleavages, one within a transmembrane segment. Cell.

[13] Wang B, Tontonoz P (2018). Liver X receptors in lipid signalling and membrane homeostasis. Nat Rev Endocrinol.

[14] Lange Y (1989). Plasma membranes contain half the phospholipid and 90% of the cholesterol and sphingomyelin in cultured human fibroblasts. J Biol Chem.

[15] Ikonen E (2008). Cellular cholesterol trafficking and compartmentalization. Nat Rev Mol Cell Biol.

[16] Mesmin B, Maxfield FR (2009). Intracellular sterol dynamics. Biochim Biophys Acta.

[17] Goldstein JL (2006). Protein sensors for membrane sterols. Cell.

[18] Chakrabarti RS (2017). Variability of cholesterol accessibility in human red blood cells measured using a bacterial cholesterol-binding toxin. Elife.

[19] Das A (2014). Three pools of plasma membrane cholesterol and their relation to cholesterol homeostasis. Elife.

[20] Gay A (2015). Switch-like responses of two cholesterol sensors do not require protein oligomerization in membranes. Biophys J.

[21] Lange Y (2004). How cholesterol homeostasis is regulated by plasma membrane cholesterol in excess of phospholipids. Proc Natl Acad Sci U S A.

[22] Demel RA (1977). The preferential interaction of cholesterol with different classes of phospholipids. Biochim Biophys Acta.

[23] Keller SL (2000). Saturated phospholipids with high melting temperatures form complexes with cholesterol in monolayers. J Phys Chem B.

[24] Lönnfors M (2011). Sterols have higher affinity for sphingomyelin than for phosphatidylcholine bilayers even at equal acyl-chain order. Biophys J.

[25] Niu SL, Litman BJ (2002). Determination of membrane cholesterol partition coefficient using a lipid vesicle-cyclodextrin binary system: effect of phospholipid acyl chain unsaturation and headgroup composition. Biophys J.

[26] Lange Y (2013). Stability and stoichiometry of bilayer phospholipid-cholesterol complexes: relationship to cellular sterol distribution and homeostasis. Biochemistry.

[27] Verkleij AJ (1973). The asymmetric distribution of phospholipids in the human red cell membrane. A combined study using phospholipases and freeze-etch electron microscopy. Biochim Biophys Acta.

[28] Radhakrishnan A, McConnell HM (2000). Chemical activity of cholesterol in membranes. Biochemistry.

[29] Infante RE, Radhakrishnan A (2017). Continuous transport of a small fraction of plasma membrane cholesterol to endoplasmic reticulum regulates total cellular cholesterol. Elife.

[30] Lange Y (2014). Essentially all excess fibroblast cholesterol moves from plasma membranes to intracellular compartments. PLoS One.

[31] Chang CCY (2000). Immunological quantitation and localization of ACAT-1 and ACAT-2 in human liver and small intestine. J Biol Chem.

[32] Suckling KE, Stange EF (1985). Role of acyl-CoA: cholesterol acyltransferase in cellular cholesterol metabolism. J Lipid Res.

[33] Chang CC (1993). Molecular cloning and functional expression of human acyl-coenzyme A:cholesterol acyltransferase cDNA in mutant Chinese hamster ovary cells. J Biol Chem.

[34] Chiang JYL (2004). Regulation of bile acid synthesis: pathways, nuclear receptors, and mechanisms. J Hepatol.

[35] Steck TL, Lange Y (2018). Transverse distribution of plasma membrane bilayer cholesterol: picking sides. Traffic.

[36] Soccio RE, Breslow JL (2004). Intracellular cholesterol transport. Arterioscler Thromb Vasc Biol.

[37] Reinisch KM, Prinz WA (2021). Mechanisms of nonvesicular lipid transport. J Cell Biol.

[38] Wu H (2018). Here, there, and everywhere: the importance of ER membrane contact sites. Science.

[39] Lipp NF (2020). Lipid exchangers: cellular functions and mechanistic links with phosphoinositide metabolism. Front Chell Dev Biol.

[40] Ikonen E, Zhou X (2021). Cholesterol transport between cellular membranes: a balancing act between interconnected lipid fluxes. Dev Cell.

[41] Ikonen E, Olkkonen VM (2023). Intracellular cholesterol trafficking. Cold Spring Harb Perspect Biol.

[42] Baumann NA (2005). Transport of newly synthesized sterol to the sterol-enriched plasma membrane occurs via nonvesicular equilibration. Biochemistry.

[43] Urbani L, Simoni RD (1990). Cholesterol and vesicular stomatitis virus G protein take separate routes from the endoplasmic reticulum to the plasma membrane. J Biol Chem.

[44] Arora A (2022). Coordination of inter-organelle communication and lipid fluxes by OSBP-related proteins. Prog Lipid Res.

[45] de Saint-Jean M (2011). Osh4p exchanges sterols for phosphatidylinositol 4-phosphate between lipid bilayers. J Cell Biol.

[46] Mesmin B (2013). A four-step cycle driven by PI4P hydrolysis directs sterol/PI(4)P exchange by the ER-Golgi tether OSBP. Cell.

[47] Godi A (2004). FAPPs control Golgi-to-cell-surface membrane traffic by binding to ARF and PtdIns(4)P. Nat Cell Biol.

[48] Lim CY (2019). ER-lysosome contacts enable cholesterol sensing by mTORC1 and drive aberrant growth signalling in Niemann-Pick type C. Nat Cell Biol.

[49] Sobajima T (2018). The Rab11-binding protein RELCH/KIAA1468 controls intracellular cholesterol distribution. J Cell Biol.

[50] Johansson M (2002). The two variants of oxysterol binding protein-related protein-1 display different tissue expression patterns, have different intracellular localization, and are functionally distinct. Mol Biol Cell.

[51] Zhao K (2020). Oxysterol-binding protein-related protein 1 variants have opposing cholesterol transport activities from the endolysosomes. Mol Biol Cell.

[52] Wang H (2019). R-loops as cellular regulators and genomic threats. Mol Cell.

[53] Woodman PG, Futter CE (2008). Multivesicular bodies: co-ordinated progression to maturity. Curr Opin Cell Biol.

[54] van der Kant R (2013). Cholesterol-binding molecules MLN64 and ORP1L mark distinct late endosomes with transporters ABCA3 and NPC1. J Lipid Res.

[55] Eden ER (2016). Annexin A1 tethers membrane contact sites that mediate ER to endosome cholesterol transport. Dev Cell.

[56] Zheng Koh DH, Saheki Y (2021). Regulation of plasma membrane sterol homeostasis by nonvesicular lipid transport. Contact (thousand oaks).

[57] Alpy F, Tomasetto C (2005). Give lipids a START: the StAR-related lipid transfer (START) domain in mammals. J Cell Sci.

[58] Tsujishita Y, Hurley JH (2000). Structure and lipid transport mechanism of a StAR-related domain. Nat Struct Biol.

[59] Clark BJ (1994). The purification, cloning, and expression of a novel luteinizing hormone-induced mitochondrial protein in MA-10 mouse Leydig tumor cells. Characterization of the steroidogenic acute regulatory protein (StAR). J Biol Chem.

[60] Alpy F (2013). STARD3 or STARD3NL and VAP form a novel molecular tether between late endosomes and the ER. J Cell Sci.

[61] Soccio RE (2002). The cholesterol-regulated StarD4 gene encodes a StAR-related lipid transfer protein with two closely related homologues, StarD5 and StarD6. Proc Natl Acad Sci U S A.

[62] Rodriguez-Agudo D (2012). ER stress increases StarD5 expression by stabilizing its mRNA and leads to relocalization of its protein from the nucleus to the membranes. J Lipid Res.

[63] Rodriguez-Agudo D (2019). StarD5: an ER stress protein regulates plasma membrane and intracellular cholesterol homeostasis. J Lipid Res.

[64] Kakiyama G (2024). StarD5 levels of expression correlate with onset and progression of steatosis and liver fibrosis. Am J Physiol Gastrointest Liver Physiol.

[65] Mesmin B (2011). STARD4 abundance regulates sterol transport and sensing. Mol Biol Cell.

[66] Iaea DB (2020). Stable reduction of STARD4 alters cholesterol regulation and lipid homeostasis. Biochim Biophys Acta Mol Cell Biol Lipids.

[67] Iaea DB (2015). STARD4 membrane interactions and sterol binding. Biochemistry.

[68] Soccio RE (2005). Differential gene regulation of StarD4 and StarD5 cholesterol transfer proteins. Activation of StarD4 by sterol regulatory element-binding protein-2 and StarD5 by endoplasmic reticulum stress. J Biol Chem.

[69] Riegelhaupt JJ (2010). Targeted disruption of steroidogenic acute regulatory protein D4 leads to modest weight reduction and minor alterations in lipid metabolism. J Lipid Res.

[70] Sandhu J (2018). Aster proteins facilitate nonvesicular plasma membrane to ER cholesterol transport in mammalian cells. Cell.

[71] Naito T (2019). Movement of accessible plasma membrane cholesterol by the GRAMD1 lipid transfer protein complex. Elife.

[72] Besprozvannaya M (2018). GRAM domain proteins specialize functionally distinct ER-PM contact sites in human cells. Elife.

[73] Begley MJ (2006). Molecular basis for substrate recognition by MTMR2, a myotubularin family phosphoinositide phosphatase. Proc Natl Acad Sci U S A.

[74] Lemmon MA (1995). Specific and high-affinity binding of inositol phosphates to an isolated pleckstrin homology domain. Proc Natl Acad Sci U S A.

[75] Trinh MN (2022). Interplay between Asters/GRAMD1s and phosphatidylserine in intermembrane transport of LDL cholesterol. Proc Natl Acad Sci U S A.

[76] Maekawa M, Fairn GD (2015). Complementary probes reveal that phosphatidylserine is required for the proper transbilayer distribution of cholesterol. J Cell Sci.

[77] Ercan B (2021). Molecular basis of accessible plasma membrane cholesterol recognition by the GRAM domain of GRAMD1b. EMBO J.

[78] Reuter MS (2017). Diagnostic yield and novel candidate genes by exome sequencing in 152 consanguineous families with neurodevelopmental disorders. JAMA Psychiatry.

[79] Santos-Cortez RLP (2018). Novel candidate genes and variants underlying autosomal recessive neurodevelopmental disorders with intellectual disability. Hum Genet.

[80] Yu L (2003). Stimulation of cholesterol excretion by the liver X receptor agonist requires ATP-binding cassette transporters G5 and G8. J Biol Chem.

[81] Xiao X (2023). Hepatic nonvesicular cholesterol transport is critical for systemic lipid homeostasis. Nat Metab.

[82] Thomas AM (2010). Genome-wide tissue-specific farnesoid X receptor binding in mouse liver and intestine. Hepatology.

[83] Acton S (1996). Identification of scavenger receptor SR-BI as a high density lipoprotein receptor. Science.

[84] Pownall HJ (2021). High-density lipoproteins, reverse cholesterol transport and atherogenesis. Nat Rev Cardiol.

[85] Payne AH, Hales DB (2004). Overview of steroidogenic enzymes in the pathway from cholesterol to active steroid hormones. Endocr Rev.

[86] Neculai D (2013). Structure of LIMP-2 provides functional insights with implications for SR-BI and CD36. Nature.

[87] Miller WL, Bose HS (2011). Early steps in steroidogenesis: intracellular cholesterol trafficking. J Lipid Res.

[88] Connelly MA (2009). SR-BI-mediated HDL cholesteryl ester delivery in the adrenal gland. Mol Cell Endocrinol.

[89] Kraemer FB (2002). Adrenal neutral cholesteryl ester hydrolase: identification, subcellular distribution, and sex differences. Endocrinology.

[90] Rigotti A (1997). A targeted mutation in the murine gene encoding the high density lipoprotein (HDL) receptor scavenger receptor class B type I reveals its key role in HDL metabolism. Proc Natl Acad Sci U S A.

[91] Meiner VL (1996). Disruption of the acyl-CoA:cholesterol acyltransferase gene in mice: evidence suggesting multiple cholesterol esterification enzymes in mammals. Proc Natl Acad Sci U S A.

[92] Cui J (2013). Estrogen synthesis and signaling pathways during aging: from periphery to brain. Trends Mol Med.

[93] Freeman EW (2010). Obesity and reproductive hormone levels in the transition to menopause. Menopause.

[94] Kautzky-Willer A (2016). Sex and gender differences in risk, pathophysiology and complications of type 2 diabetes mellitus. Endocr Rev.

[95] Xiao X (2024). Aster-B-dependent estradiol synthesis protects female mice from diet-induced obesity. J Clin Invest.

[96] Brown AJ (2021). Oxysterols: from physiological tuners to pharmacological opportunities. Br J Pharmacol.

[97] de Aguiar Vallim TQ (2013). Pleiotropic roles of bile acids in metabolism. Cell Metab.

[98] Banerjee R (2024). The nonvesicular sterol transporter Aster-C plays a minor role in whole body cholesterol balance. Front Physiol.

[99] Horton JD (1998). Regulation of sterol regulatory element binding proteins in livers of fasted and refed mice. Proc Natl Acad Sci U S A.

[100] Moller L (2008). Fasting in healthy subjects is associated with intrahepatic accumulation of lipids as assessed by 1H-magnetic resonance spectroscopy. Clin Sci (Lond).

[101] Goldstein JL, Brown MS (1974). Binding and degradation of low density lipoproteins by cultured human fibroblasts. Comparison of cells from a normal subject and from a patient with homozygous familial hypercholesterolemia. J Biol Chem.

[102] Anderson RGW (1977). Role of the coated endocytic vesicle in the uptake of receptor-bound low density lipoprotein in human fibroblasts. Cell.

[103] Anderson RA, Sando GN (1991). Cloning and expression of cDNA encoding human lysosomal acid lipase/cholesteryl ester hydrolase. Similarities to gastric and lingual lipases. J Biol Chem.

[104] Pfisterer SG (2016). LDL-cholesterol transport to the endoplasmic reticulum: current concepts. Curr Opin Lipidol.

[105] Kanerva K (2013). LDL cholesterol recycles to the plasma membrane via a Rab8a-Myosin5b-actin-dependent membrane transport route. Dev Cell.

[106] Raiborg C (2015). ER-endosome contact sites: molecular compositions and functions. EMBO J.

[107] Tabas I (1988). Acyl coenzyme A:cholesterol acyl transferase in macrophages utilizes a cellular pool of cholesterol oxidase-accessible cholesterol as substrate. J Biol Chem.

[108] Brasaemle DL, Attie AD (1990). Rapid intracellular transport of LDL-derived cholesterol to the plasma membrane in cultured fibroblasts. J Lipid Res.

[109] Lange Y (1997). The fate of cholesterol exiting lysosomes. J Biol Chem.

[110] Kwon HJ (2009). Structure of N-terminal domain of NPC1 reveals distinct subdomains for binding and transfer of cholesterol. Cell.

[111] Qian H (2020). Structural basis of low-pH-dependent lysosomal cholesterol egress by NPC1 and NPC2. Cell.

[112] Takahashi K (2021). ORP2 couples LDL-cholesterol transport to FAK activation by endosomal cholesterol/PI(4,5)P_2_ exchange. EMBO J.

[114] Trinh MN (2020). Last step in the path of LDL cholesterol from lysosome to plasma membrane to ER is governed by phosphatidylserine. Proc Natl Acad Sci U S A.

[115] Xiao X (2021). Selective Aster inhibitors distinguish vesicular and nonvesicular sterol transport mechanisms. Proc Natl Acad Sci U S A.

[116] Mansbach CM, Siddiqi SA (2010). The biogenesis of chylomicrons. Annu Rev Physiol.

[117] Nguyen TM (2012). Cholesterol esterification by ACAT2 is essential for efficient intestinal cholesterol absorption: evidence from thoracic lymph duct cannulation. J Lipid Res.

[118] Zhang J (2012). Tissue-specific knockouts of ACAT2 reveal that intestinal depletion is sufficient to prevent diet-induced cholesterol accumulation in the liver and blood. J Lipid Res.

[119] Altmann SW (2004). Niemann-Pick C1 Like 1 protein is critical for intestinal cholesterol absorption. Science.

[120] Ferrari A (2023). Aster-dependent nonvesicular transport facilitates dietary cholesterol uptake. Science.

[121] Hu M (2021). Structural insights into the mechanism of human NPC1L1-mediated cholesterol uptake. Sci Adv.

[122] Huang CS (2020). Cryo-EM structures of NPC1L1 reveal mechanisms of cholesterol transport and ezetimibe inhibition. Sci Adv.

[123] Long T (2021). Structures of dimeric human NPC1L1 provide insight into mechanisms for cholesterol absorption. Sci Adv.

[124] Li PS (2014). The clathrin adaptor Numb regulates intestinal cholesterol absorption through dynamic interaction with NPC1L1. Nat Med.

[125] Xie C (2012). Ezetimibe blocks the internalization of NPC1L1 and cholesterol in mouse small intestine. J Lipid Res.

[126] Zhang YY (2018). A *LIMA1* variant promotes low plasma LDL cholesterol and decreases intestinal cholesterol absorption. Science.

